# Comparison of properties determined using electromechanical assessment (Arthro-BST™) with macroscopic and histological properties in symptomatic human articular cartilage of the hip

**DOI:** 10.1186/s13075-021-02611-x

**Published:** 2021-08-31

**Authors:** Taku Ukai, Masato Sato, Shiho Wasai, Takumi Takahashi, Haruka Omura, Masahiko Watanabe

**Affiliations:** 1 Department of Orthopedic Surgery, Surgical Science, Tokai University School of Medicine, 143 Shimokasuya, Bohseidai, Isehara, Kanagawa 259-1193 Japan; 2 Center for Musculoskeletal Innovative Research and Advancement (C-MiRA), Tokai University Graduate School, Shimokasuya, Isehara, Kanagawa 259-1193 Japan

**Keywords:** Osteoarthritis, Arthro-BST, ICRS classification, Modified Mankin score, OARSI histological system

## Abstract

**Background:**

Cartilage degeneration is assessed using various methods. Although macroscopic evaluation can directly measure cartilage degeneration, it cannot accurately assess cartilage properties. Histological examination is one of the most accurate methods for evaluating cartilage degeneration. However, it is invasive and requires collection of cartilage tissue. In contrast, the Arthro-BST™ probe can assess cartilage properties noninvasively. This study aimed to evaluate the effectiveness of the Arthro-BST in assessing cartilage degeneration by comparing macroscopic (International Cartilage Repair Society [ICRS] classification) and histological evaluations (modified Mankin score and Osteoarthritis Research Society International [OARSI] histological grade).

**Methods:**

Fourteen femoral heads were excised from 13 patients during surgery to treat hip osteoarthritis or femoral fracture. The ICRS score was used for macroscopic evaluation of cartilage degeneration. The Arthro-BST was applied at sites matching the areas of cartilage damage. The sites assessed using the ICRS classification and Arthro-BST were evaluated histologically (modified Mankin score and OARSI histological grade), and these were compared with the Arthro-BST results.

**Results:**

The ICRS classification identified significant differences between grades 1 and 3 (*p* < 0.01), between grades 1 and 4 (*p* < 0.01), between grades 2 and 3 (*p* < 0.01), and between grades 2 and 4 (*p* < 0.01). Significant correlations were observed between the Arthro-BST results and the ICRS score, modified Mankin score (structure, cellularity, matrix staining, total score), and OARSI histological grade.

**Conclusions:**

In the assessment of hip osteoarthritis, the Arthro-BST results correlated with those of macroscopic and histological evaluations. The Arthro-BST is useful for assessing hip osteoarthritis and may be helpful for noninvasive assessment of cartilage degeneration.

**Supplementary Information:**

The online version contains supplementary material available at 10.1186/s13075-021-02611-x.

## Background

Osteoarthritis (OA) is a common form of arthritis among older people, and the number of patients with OA is increasing worldwide. Although OA is not a life-threatening disease, it affects daily activities and quality of life. Cartilage is one of the most difficult tissues to regenerate because of its avascular nature. Therefore, detecting early degeneration of articular cartilage is necessary for the prevention and treatment of OA. Various diagnostic methods, such as radiography [1], macroscopic imaging [2], magnetic resonance imaging (MRI) [3], and histological evaluation [4, 5], are used for evaluating OA.

Although radiography is the most frequently used technique for evaluating OA through assessment of joint space and osteophyte formation, this method cannot be used to evaluate the macroscopic changes and properties of articular cartilage. Arthroscopy is used widely for treating cartilage degeneration, and although it can be used to assess cartilage degeneration directly, it cannot be used to evaluate cartilage properties. MRI has recently been used for assessing cartilage degeneration. We have focused on the noninvasive assessment of cartilage degeneration and have reported the usefulness of MRI for assessing knee OA using T2 mapping and diffusion tensor imaging [3]. Although MRI can be used to assess cartilage degeneration noninvasively, it is difficult to assess cartilage degeneration when the cartilage damage is of a mixed nature. Histological evaluation can be used to assess cartilage degeneration accurately, but it involves the destruction of normal cartilage.

To overcome these disadvantages, nondestructive devices have been developed to assess cartilage degeneration [6–21]. We explored the literature on the assessment of cartilage degeneration that used laser-induced photoacoustic measurement (LIPA) instruments [21]. These devices can assess cartilage degeneration without the need to collect tissue samples. We have previously compared LIPA with histological evaluation and reported that LIPA can be used to assess cartilage degeneration and viscoelastic properties [21].

Articular cartilage contains proteoglycans that have electromechanical properties [22]. Interstitial water contains positive mobile ions, such as Na^+^ and K^+^, which balance the fixed negative electric charge from proteoglycans [22]. The mechanical compression of articular cartilage generates streaming potentials induced by water flowing out from articular cartilage [23], and these streaming potentials reflect cartilage integrity and degeneration [24–27]. Taking advantage of this property, the Arthro-BST instrument (Biomomentum, Laval, QC, Canada) was invented for assessing the streaming potentials of articular cartilage. This apparatus is used during arthroscopy and induces streaming potentials using 37 microelectrodes with the help of the spherical indenter of the tip. The effectiveness of the Arthro-BST for assessing the properties of articular cartilage of the knee has been reported previously [28–31]. However, no study has evaluated the effectiveness of the Arthro-BST for assessing the hip by evaluating the macroscopic and histological properties simultaneously.

This study aimed to evaluate the effectiveness of the Arthro-BST for evaluating the hip by comparing its findings with macroscopic findings. We used the International Cartilage Repair Society (ICRS) classification system [2] and histological findings such as the modified Mankin histological score [32] and Osteoarthritis Research Society International (OARSI) histopathology assessment system [5].

## Methods

### Sample source

Tissue was obtained from patients who had been diagnosed with OA or femoral fracture of the hip and who underwent total hip arthroplasty or bipolar hip arthroplasty at the authors’ institution. After resection of the femoral head of the hip, 14 femoral heads of 13 patients were evaluated (Table 1). This study was performed after approval from the research review committee at the author’s institution (approval number: 18R-187). All patients provided written informed consent.

**Table 1:** Patient demographics

Patient number	Age (years)	Sex	Body mass index (kg/m^2^)	Diagnosis	Kellgren–Lawrence classification
1	72	F	26.2	Osteoarthritis	3
2	57	F	24.4	Osteoarthritis	3
3	65	F	22	Osteoarthritis	3
4	60	F	34.9	Osteoarthritis	4
5	83	F	18.6	Osteoarthritis	4
6	74	F	23.7	Osteoarthritis	4
7	82	F	17.2	Femoral neck fracture	1
8	67	F	26	Osteoarthritis	3
9	84	F	23	Femoral neck fracture	1
10	64	F	23.1	Osteoarthritis	4
11	54	M	24.8	Osteoarthritis	4
12	70	F	27.4	Osteoarthritis	4
13	71	F	23.2	Femoral neck fracture	1
14	55	F	24.4	Osteoarthritis	3

### Assessment procedure

After resection of the femoral head, cartilage lesions were assessed macroscopically using the ICRS classification [2]. The same sites of cartilage lesions were assessed histologically using the modified Mankin score [32] and OARSI grade [5].

### Macroscopic assessment

**Fig. 1:**
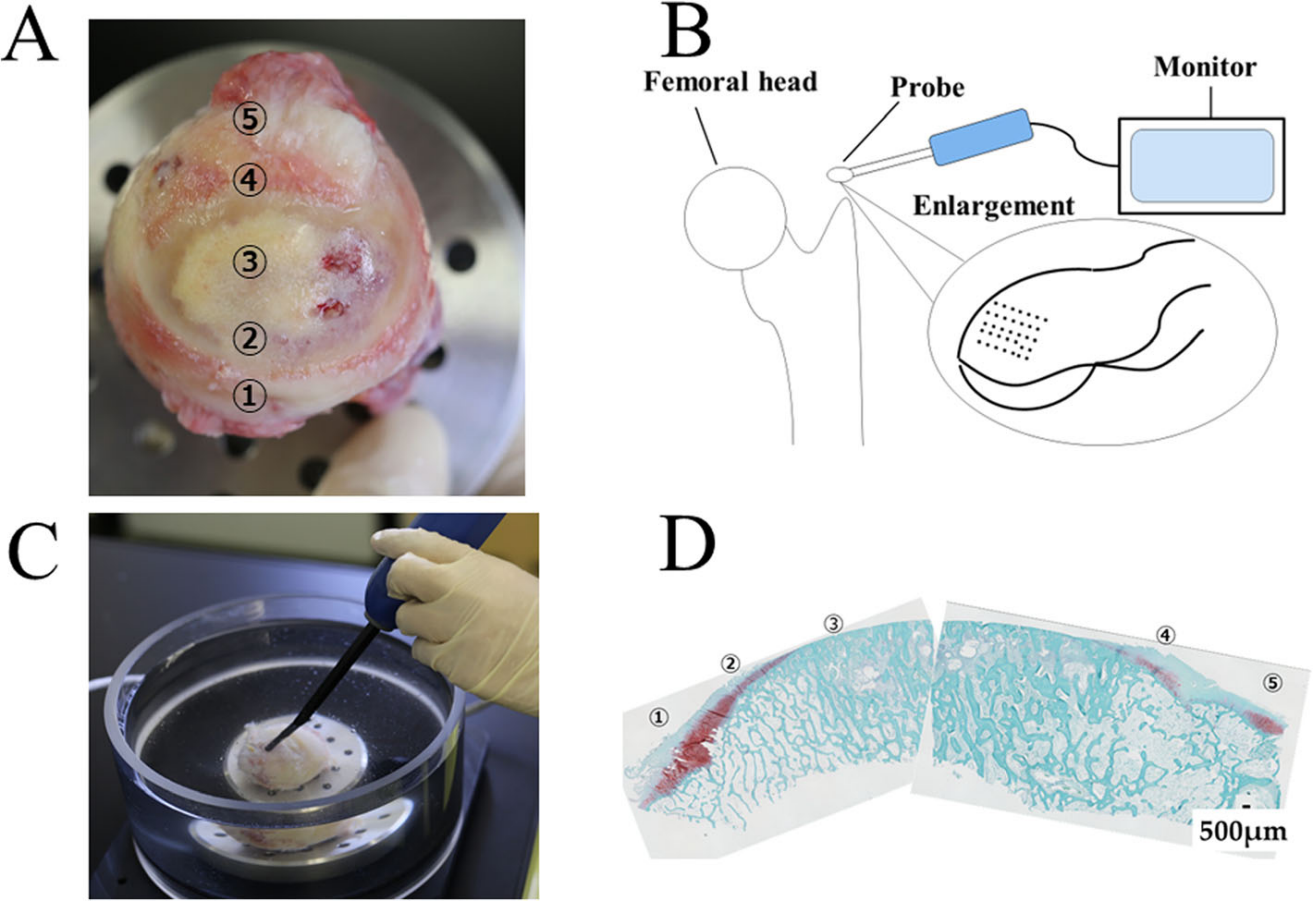
Cartilage tissue assessment. **A** The ICRS classification was used to assess the cartilage of the femoral head (see Table 1 for the definition of the grades). **B** Schematic drawing of the Arthro-BST. The Arthro-BST induces streaming potentials using 37 microelectrodes. **C** The Arthro-BST was used to assess the same points assessed by the ICRS classification. **D** Cartilage sections were made by cutting perpendicular to the same points assessed using the ICRS classification and Arthro-BST

After dissection of the femoral head, the assessment points were marked with the help of photographs (Fig. 1). Two experienced orthopedic surgeons separately performed a macroscopic assessment of 14 femoral heads. Sixty-one locations of the femoral head were used to assess the ICRS classification (Table 2). Each femoral head was assessed for cartilage degeneration on the day of surgery.

**Table 2:** ICRS classification as described by Mainil-Varlet et al. [2]

Grade	Property
1	Superficial lesions, fissures and cracks, soft indentation
2	Defects that extend to less than 50% in depth
3	Defects that extend to more than 50% in depth
4	Complete loss of cartilage thickness, bone only

### Electromechanical assessment

The Arthro-BST measures streaming potentials generated during rapid compression of the articular cartilage with an array of 37 microelectrodes lying on a hemispherical indenter (effective radius of the tip = 3.18 mm, 5 microelectrodes/mm^2^) [29]. The device measures a quantitative parameter (QP), which corresponds to the number of microelectrodes in contact with the cartilage when the sum of all electrode potentials reaches 100 mV [30]. A high QP indicates strong electromechanical properties and normal cartilage, and a low QP indicates weak electromechanical properties and degenerated cartilage.

The resected femoral heads were placed onto a cylindrical platform and assembled into a testing chamber using screws. A single electromechanical measurement was performed manually at each position of the grid using the Arthro-BST. Measurements were performed on the day of surgery (Fig. 1).

### Histological assessment

Twenty-five samples were used for histological assessment. Two experienced orthopedic surgeons separately performed a histological assessment of each sample. The tissue samples were cut into sections by making cuts perpendicular to the cartilage surface and then fixed in 4% paraformaldehyde for 1 month. After decalcification for 2 months using distilled water (pH 7.4) containing 10% ethylenediaminetetraacetic acid, the tissue was embedded in paraffin wax and sectioned perpendicularly through the center of the cartilage damage. Each section was stained with Safranin O dye for histological evaluation of glycosaminoglycans [33]. For histological assessment, sections stained with Safranin O were assessed using the modified Mankin score [32] and the OARSI histopathology assessment system (grade 0, surface intact, cartilage morphology intact; grade 1, surface intact; grade 2, surface discontinuity; grade 3, vertical fissures (clefts); grade 4, erosion; grade 5, denudation; grade 6, deformation) [5]. Two orthopedic surgeons of the Japanese Orthopedic Association independently performed the histological assessment. The modified Mankin score was determined by adding the scores of the parameters (structure, cellularity, matrix staining, and tidemark integrity), with 0 as the lowest score and 15 as the highest score (Table 3).

**Table 3:** Modified Mankin score for evaluation of articular cartilage degeneration as described by Henson and Vincent [32]

Score	Structure	Cellularity	Matrix staining	Tidemark integrity
Score 0	Smooth surface	Normal arrangement	Normal staining	Normal and intact
	Normal appearance			
Score 1	Roughened surface	Clustering in the superficial layer or loss of cells up to 10%	Slight loss of stain	Disrupted
	Single crack or area of delamination			
Score 2	Multiple cracks	Disorganization or loss up to 25%	Moderate loss of stain	
	Moderate delamination			
Score 3	Fragmentation in cartilage or severe delamination	Cell rows absent or loss up to 50%	Severe loss of stain	
Score 4	Loss of fragments	Very few cells present	No stain present	
Score 5	Complete erosion to tidemark			
Score 6	Erosion beyond tidemark			

### Statistical analysis

Kruskal–Wallis followed by the Bonferroni post hoc test was used to compare the ICRS grade and Arthro-BST result (QP). Spearman’s rank correlational analysis was used to identify significant relationships between the Arthro-BST QP and the ICRS grade, modified Mankin score, and OARSI histological grade. Interobserver reliability was tested using intraclass correlation coefficients (ICCs), and their 95% confidence intervals (CIs) were used to assess the reliability of the macroscopic and histological assessments. All tests were performed at a significance level of *p* < 0.05. Analyses were performed using IBM SPSS Statistics for Windows version 26 (IBM Corp., Armonk, NY, USA).

## Results

### Comparison between the Arthro-BST and the macroscopic assessment results

**Fig. 2:**
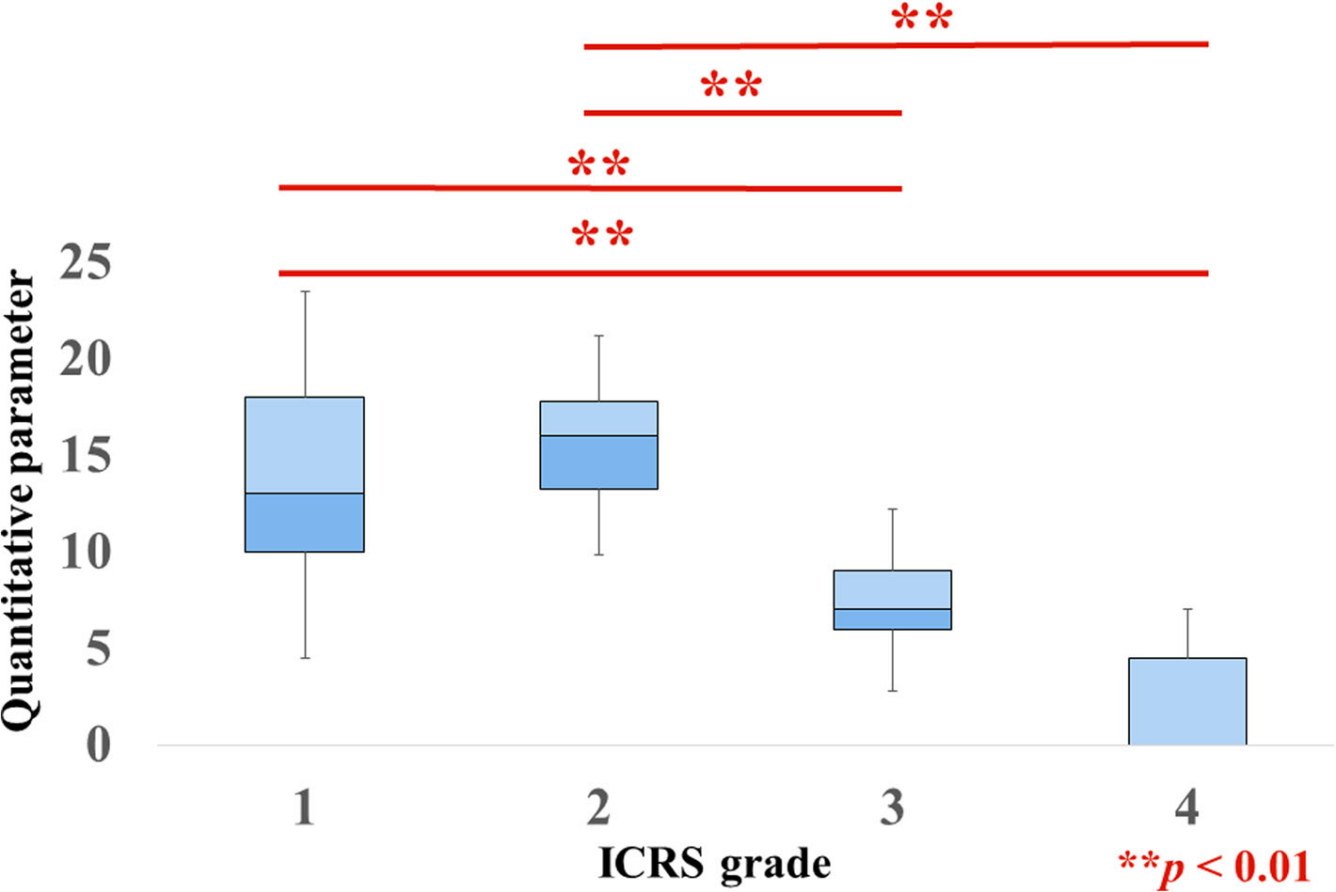
Arthro-BST assessment and ICRS classification. The QP decreased as the ICRS grade increased. Significant differences were observed for ICRS grades 1 and 3 (*p* < 0.01), grades 1 and 4 (*p* < 0.01), grades 2 and 3 (*p* < 0.01), and grades 2 and 4 (*p* < 0.01)

The ICRS grades of the cartilage lesion for all samples were grade 1 (*n* = 23), grade 2 (*n* = 18), grade 3 (*n* = 13), and grade 4 (*n* = 7). The interobserver reliability of the ICRS classification was 0.973 (95% CI, 0.956–0.984) (Additional file 1). The QPs for the grades were as follows: grade 1, 14.1 ± 5.5; grade 2, 15.6 ± 3.4; grade 3, 7.8 ± 3.2; and grade 4, 2 ± 2.5. Significant differences were observed between grades 1 and 3 (*p* < 0.01), between grades 1 and 4 (*p* < 0.01), between grades 2 and 3 (*p* < 0.01), and between grades 2 and 4 (*p* < 0.01) (Fig. 2).

### Relationship between the Arthro-BST QP and the macroscopic assessment

**Fig. 3:**
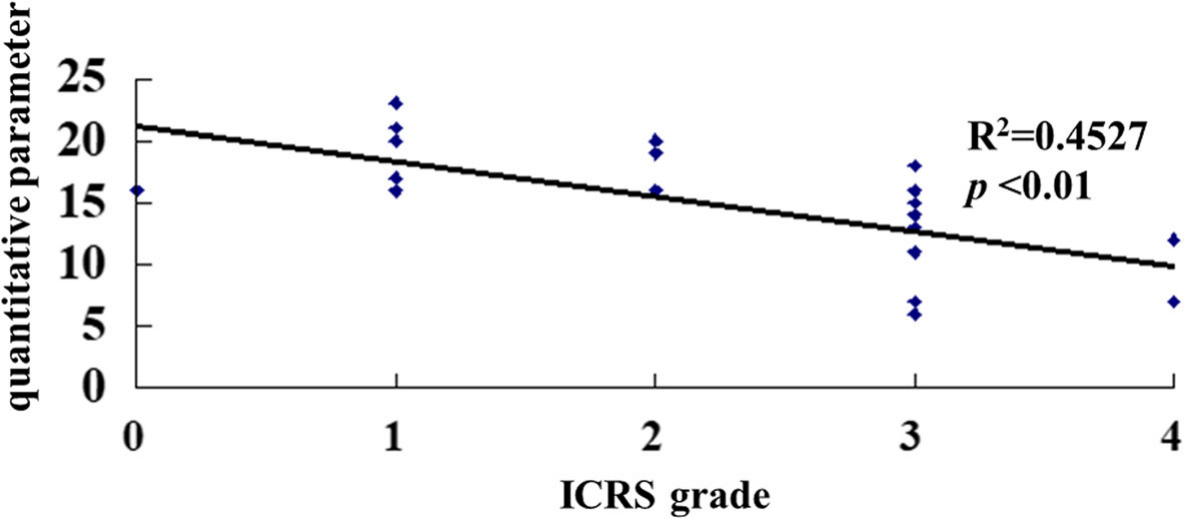
Scatter plot of the correlation between the Arthro-BST results and the ICRS classification. Each diamond represents the QP according to the ICRS grade in an individual hip. A significant strong correlation was observed between the Arthro-BST and the ICRS classification (*R*^2^ = 0.4527)

The QP decreased as the ICRS grade increased, and the correlation between these variables was significant (*p* < 0.01) (Fig. 3).

### Relationship between the Arthro-BST and the histological assessment

#### Modified Mankin score: structure

**Fig. 4:**
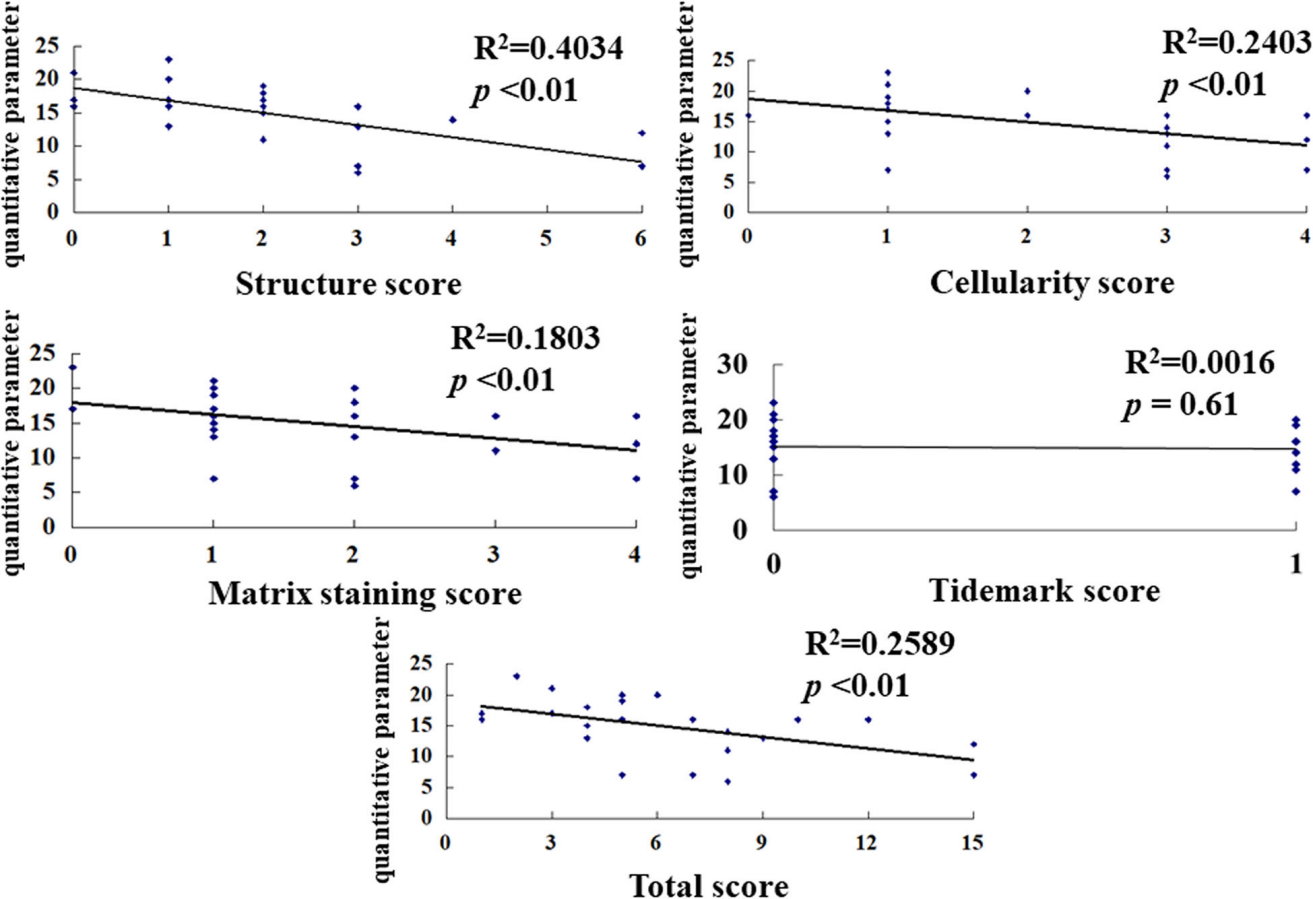
Scatter plot of the correlations between the Arthro-BST and the modified Mankin score. Each diamond represents the QP values for the structure, cellularity, matrix staining, tidemark, and total score in an individual hip. The Arthro-BST result correlated significantly with structure (*R*^2^ = 0.4034), cellularity (*R*^2^ = 0.2403), matrix staining (*R*^2^ = 0.1803), and total score (*R*^2^ = 0.2589)

The values for the structure parameter of the modified Mankin score were 0 (*n* = 3), 1 (*n* = 8), 2 (*n* = 6), 3 (*n* = 5), 4 (*n* = 1), 5 (*n* = 0), and 6 (*n* = 2). The interobserver reliability of the score for structure was 0.877 (95% CI, 0.725–0.946) (Additional file 2). The QPs for the scores were as follows: score 0, 18 ± 2.6; score 1, 17.9 ± 3.3; score 2, 16 ± 2.6; score 3, 9.8 ± 4.4; score 4, 14; and score 6, 9.5 ± 3.5. The QP decreased as the structure score increased, and the correlation between these was significant (*p* < 0.01) (Fig. 4).

#### Cellularity

The values for the cellularity parameter of the modified Mankin score were 0 (*n* = 1), 1 (*n* = 10), 2 (*n* = 5), 3 (*n* = 6), and 4 (*n* = 3). The interobserver reliability of the cellularity score was 0.9 (95% CI, 0.775–0.956) (Additional file 2). The QPs for the scores were as follows: score 0, 16; score 1, 16.7 ± 4.4; score 2, 17.6 ± 2.2; score 3, 11.1 ± 4; and score 4, 11.7 ± 4.5. The QP decreased as the cellularity score increased, and the correlation between these was significant (*p* < 0.01) (Fig. 4).

#### Matrix staining

The values for the matrix staining parameter of the modified Mankin score were 0 (*n* = 2), 1 (*n* = 10), 2 (*n* = 8), 3 (*n* = 2), and 4 (*n* = 3). The interobserver reliability for the matrix staining score was 0.874 (95% CI, 0.717–0.944) (Additional file 2). The QPs for the score were as follows: score 0, 20 ± 4.2; score 1, 15.9 ± 4; score 2, 14 ± 5; score 3, 13.5 ± 3.5; and score 4, 11.7 ± 4.5. The QP decreased as the matrix staining score increased, and the correlation between these was significant (*p* < 0.01) (Fig. 4).

#### Tidemark

The values for the tidemark parameter of the modified Mankin score were 0 (*n* = 15) and 1 (*n* = 10). The interobserver reliability for the tidemark score was 0.868 (95% CI, 0.704–0.942) (Additional file 2). The QPs for the scores were as follows: score 0, 15.1 ± 5.1; score 1, 14.7 ± 3.9. The correlation between the QP and the tidemark score was not significant (Fig. 4).

#### Total score

**Fig. 5:**
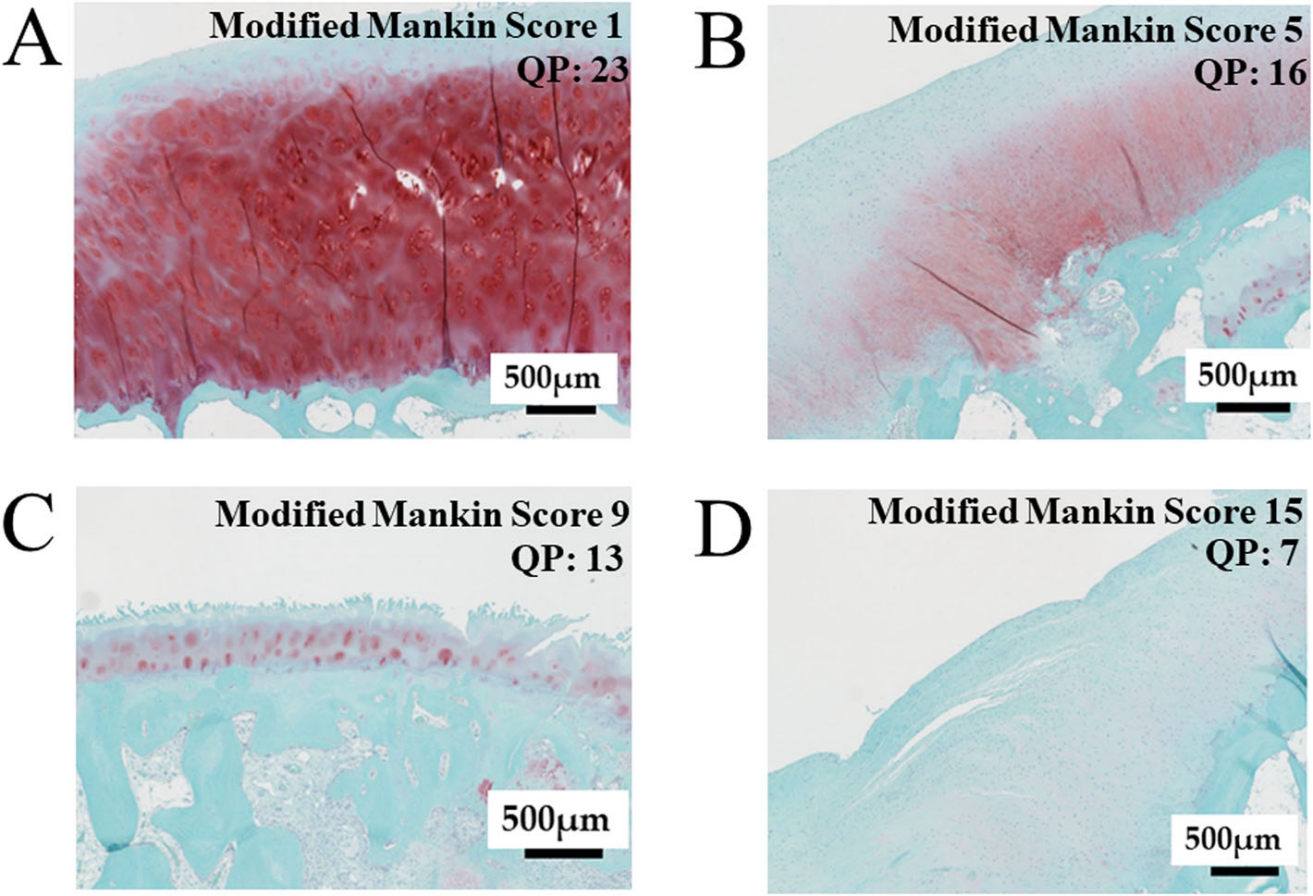
Histological assessment: representative Safranin O-stained sections for four parameters of the modified Mankin score and the corresponding QP. **A** The modified Mankin score of the cartilage section was 1 (structure, 1; cellularity, 0; matrix staining, 0; tidemark, 0; total, 1). The QP of the same point was 23. **B** The modified Mankin score of the cartilage section was 5 (structure, 0; cellularity, 2; matrix staining, 2; tidemark, 1; total, 5). The QP of the same point was 16. **C** The modified Mankin score of the cartilage section was 9 (structure, 4; cellularity, 3; matrix staining, 2; tidemark, 0; total, 9). The QP of the same point was 13. **D** The modified Mankin score of the cartilage section was 15 (structure, 6; cellularity, 4; matrix staining, 4; tidemark, 1; total, 15). The QP of the same point was 7

The interobserver reliability of the total score was 0.93 (95% CI, 0.844–0.969) (Additional file 2). The QP decreased as the total modified Mankin score increased, and the correlation between these was significant (*p* < 0.01) (Figs. 4 and 5).

#### OARSI histological grade

**Fig. 6:**
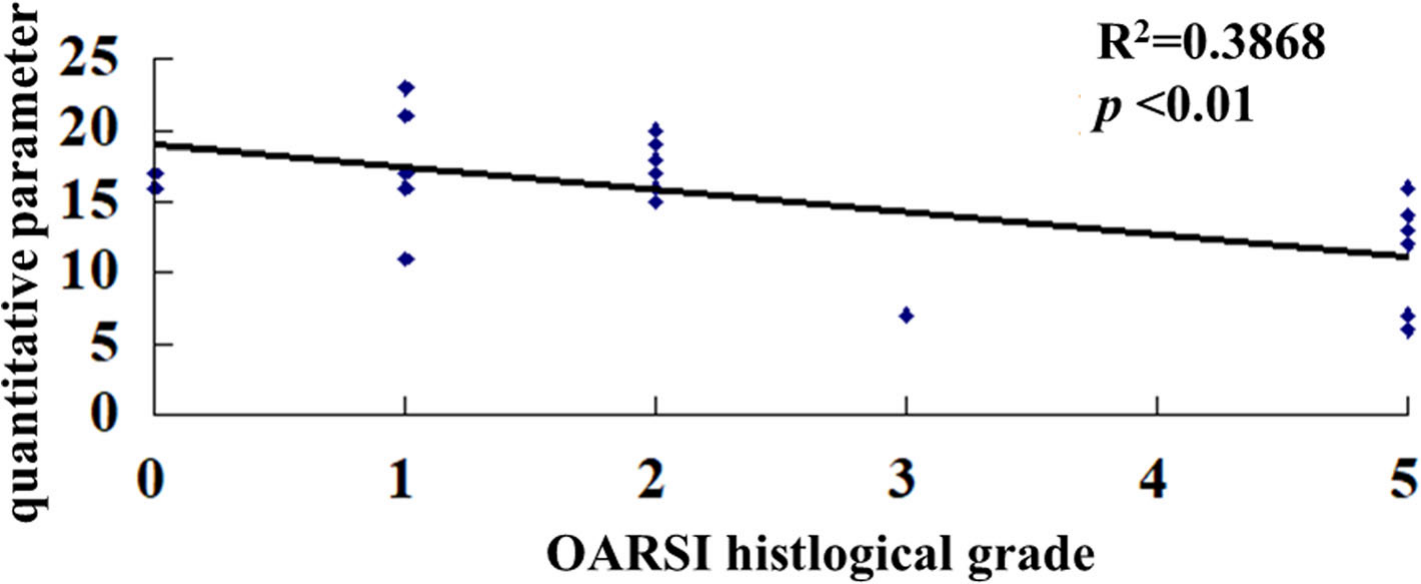
Scatter plot of the correlation between the Arthro-BST results and the OARSI histopathological score. Each diamond represents the QP according to the OARSI grade in an individual hip. A significant correlation was observed between the Arthro-BST results and the OARSI histopathological score (*R*^2^ = 0.3868)

The OARSI grades of cartilage lesions were as follows: grade 0 (*n* = 2), grade 1 (*n* = 6), grade 2 (*n* = 8), grade 3 (*n* = 1), grade 4 (*n* = 0), and grade 5 (*n* = 8). The interobserver reliability for the OARSI histological grade was 0.903 (95% CI, 0.782–0.957) (Additional file 3). The QPs for the grades were as follows: grade 0, 16.5 ± 0.7; grade 1, 17.3 ± 4.2; grade 2, 17.6 ± 1.9; grade 3, 7; and grade 4, 11 ± 3.8. The QP decreased as the OARSI grade increased, and the correlation between these was significant (*p* < 0.01) (Fig. 6).

## Discussion

Our results demonstrated that the Arthro-BST can distinguish between ICRS grades 1 and 3, between grades 1 and 4, between grades 2 and 3, and between grades 2 and 4. There was a significant correlation between the Arthro-BST QP and the ICRS grades as well as between the Arthro-BST QP and the modified Mankin scores (structure, cellularity, matrix staining, total score) and OARSI histological grade.

Various methods are used for the evaluation of cartilage degeneration. MRI has recently been used for the evaluation of OA. We have previously reported that Outerbridge grades correlate with MRI variables such as diffusion tensor imaging and T2 mapping [3]. Although the biggest advantage of MRI is its ability to assess cartilage degeneration noninvasively, it is not capable of assessing cartilage properties; hence, other methods that can evaluate cartilage properties without the need to sample cartilage tissue are needed.

Some studies have reported that the Arthro-BST QP correlates with the ICRS grade [29, 30, 34, 35] and Mankin score [29, 30]. Although the Arthro-BST could not distinguish ICRS grade 1 from 2 and grade 3 from 4 in our results, the QP correlated significantly with the ICRS grade. Similarly, Hadjab et al. [28] reported that the QP did not correlate significantly between ICRS grades 0 and 2, which seems reasonable because the ICRS grading system cannot distinguish small differences in cartilage damage [36] and because tissues affected by OA can have lesions with mixed grades of cartilage degeneration. We did not measure cartilage thickness in this study, and we found no significant differences between ICRS grades 1 and 2 or between grades 3 and 4. However, a comparison of the Arthro-BST results and the ICRS score is important. The Arthro-BST must be used under arthroscopy because the embedded sensors must be in direct contact with the cartilage. Although the Arthro-BST could not distinguish between ICRS grades 1 and 2 or between grades 3 and 4 in our study, we consider that this apparatus would be useful for hip OA because it can distinguish early OA (ICRS grade 1) from moderate OA (ICRS grade 2), early OA from severe OA (ICRS grade 3 or 4), and moderate OA from severe OA.

Cartilage tissue is classified into articular or hyaline cartilage and fibrous cartilage. Healthy articular cartilage comprises mainly hyaline cartilage, fibrous cartilage comprises mainly type I collagen, and hyaline cartilage comprises mainly type II collagen. Fibrous cartilage is frequently seen after injury and in degenerated cartilage tissue. The deposition of fibrous cartilage in place of articular cartilage is an inferior change that can lead to secondary OA. Distinguishing between fibrous and hyaline cartilage is difficult when using only the ICRS classification, but assessing the composition of regenerative tissue is necessary to prevent secondary OA. Chondrocyte proliferation appears during the early stages of OA [37], and the proteoglycan content decreases before collagen content decreases [37–39]. These changes induce fibrillation and fissures, which affect the mechanical strength of articular cartilage [40, 41]. Therefore, it is important to determine whether the Arthro-BST results correlate with the histological data.

As for the results of the histological assessment, some authors have reported that the QP correlates significantly with the total Mankin score [29, 30]. The Mankin score includes five parameters (structure, cellularity, matrix staining, tidemark, total score), and most reports have assessed only the QP and total score. We found that the total score and other parameters correlated significantly with the QP (Fig. 4). We have previously studied the effectiveness of LIPA [21]. LIPA is based on the use of photoacoustic waves and can assess viscoelastic properties without sampling cartilage tissue. We previously reported that the LIPA results correlated strongly with the ICRS grade, although we found no correlation between the LIPA results and the overall Mankin score [21]. Compared with LIPA, the Arthro-BST QP had stronger correlations with the total score as well as with structure, cellularity, and matrix staining. Therefore, we believe that the Arthro-BST is preferable to the LIPA for assessing cartilage degeneration.

Pritzker et al. [5] were the first to report the OARSI histological system. Some studies have reported excellent correlations between the Mankin score and the OARSI histological grade [42–46]. Compared with the modified Mankin score, the OARSI histological grade focuses more on structural parameters. The Mankin score does not have a staging component [42] and has limited usefulness for assessing mild and moderate OA [47]. In contrast, the OARSI system can distinguish between early and moderate OA [2]. Subchondral bone changes occur during the first stage of OA [48, 49], and these changes affect the pathogenesis of OA [50–53]. Finnilä et al. [54] reported a high correlation between the OARSI grade and subchondral plate thickness. Therefore, we used both histological scores in this study to assess each stage of OA accurately. We found a significant correlation between the QP and the OARSI histological grade and that the Arthro-BST was able to assess all grades of OA. Therefore, we believe that the Arthro-BST is useful for the simultaneous evaluation of macroscopic and cartilage properties. This device may also help in decision-making about the best method for cartilage regeneration and for assessing regenerating tissue.

There are some limitations to this study. First, some authors have already reported an association between the Arthro-BST results and the measures of OA. However, some of these authors have used cartilage from goats [31] or from human knees for evaluation [27–30]. To our knowledge, no studies have reported on the associations between the Arthro-BST results and OA measures in human cartilage of the hip. In addition, other authors have used human cartilage that underwent freeze–thaw cycles, which may have influenced the macroscopic appearance and histological properties of the cartilage tissue. Second, the assessments of the ICRS classification score, modified Mankin score, and OARSI histological score varied somewhat, which may have affected the results. However, the assessments were performed by two different evaluators, and all ICCs were > 0.7, which indicates high reproducibility.

## Conclusions

The Arthro-BST can distinguish between ICRS grades 1 and 3, grades 1 and 4, grades 2 and 3, and grades 1 and 3. The Arthro-BST findings correlated with the macroscopic and histological assessment results. This apparatus may be helpful for performing macroscopic and histological assessments simultaneously, which may be useful for the noninvasive diagnosis of OA.

## Supplementary Information

**Additional file 1.** Individual scoring data of the ICRS classification.

**Additional file 2.** Individual scoring data of the modified Mankin score.

**Additional file 3.** Individual scoring data of the OARSI histopathological system.

## Data Availability

The datasets used and/or analyzed during the current study are available from the corresponding author on reasonable request.

## Abbreviations

CI: Confidence interval
ICC: Intraclass correlation coefficient
ICRS: International Cartilage Repair Society
LIPA: Laser-induced pulse acoustic
MRI: Magnetic resonance imaging
OA: Osteoarthritis
OARSI: Osteoarthritis Research Society International
QP: Quantitative parameter
